# Polymer-Antimicrobial Peptide Constructs with Tailored Drug-Release Behavior

**DOI:** 10.3390/pharmaceutics15020406

**Published:** 2023-01-25

**Authors:** Robert Pola, Matěj Vícha, Jiří Trousil, Eliška Grosmanová, Michal Pechar, Anna Rumlerová, Martin Studenovský, Emilie Kučerová, Pavel Ulbrich, Barbora Vokatá, Tomáš Etrych

**Affiliations:** 1 Institute of Macromolecular Chemistry, Czech Academy of Sciences, Heyrovského nám. 2, 162 00 Prague, Czech Republic; 2 Department of Biochemistry and Microbiology, University of Chemistry and Technology Prague, Technická 3, 166 28 Prague, Czech Republic

**Keywords:** antimicrobial peptides, HPMA copolymers, bacteria, drug delivery

## Abstract

Microbial resistance is one of the main problems of modern medicine. Recently, antimicrobial peptides have been recognized as a novel approach to overcome the microbial resistance issue, nevertheless, their low stability, toxicity, and potential immunogenic response in biological systems have limited their clinical application. Herein, we present the design, synthesis, and preliminary biological evaluation of polymer-antibacterial peptide constructs. The antimicrobial GKWMKLLKKILK-NH_2_ oligopeptide (PEP) derived from halictine, honey bee venom, was bound to a polymer carrier via various biodegradable spacers employing the pH-sensitive or enzymatically-driven release and reactivation of the PEP’s antimicrobial activity. The antibacterial properties of the polymer-PEP constructs were assessed by a determination of the minimum inhibitory concentrations, followed by fluorescence and transmission electron microscopy. The PEP exerted antibacterial activity against both, gram-positive and negative bacteria, via disruption of the bacterial cell wall mechanism. Importantly, PEP partly retained its antibacterial efficacy against *Staphylococcus epidermidis*, *Escherichia coli*, and *Acinetobacter baumanii* even though it was bound to the polymer carrier. Indeed, to observe antibacterial activity similar to the free PEP, the peptide has to be released from the polymer carrier in response to a pH decrease. Enzymatically-driven release and reactivation of the PEP antimicrobial activity were recognized as less effective when compared to the pH-sensitive release of PEP.

## 1. Introduction

Bacterial diseases continue to threaten human and animal health globally despite the development of new antibiotics. The success of the therapy is challenged mostly by the development of microbial resistance and tolerance, the formation of biofilms, and the intracellular localization of certain clinically important pathogens. Such resistant bacterial strains may cause life-threatening infections, such as pneumonia or tuberculosis (TB). TB, a severe respiratory disease caused by *Mycobacterium tuberculosis* (*M.tb.*) with growing resistance to traditional treatment, is one of the major threats to public health causing 1.5 million deaths annually [1,2]. *Staphylococcus aureus* (*S.a*.), causing severe skin and blood-stream infections, and *Pseudomonas aeruginosa* (*P.a*.), causing hospital-acquired infections and pneumonia in patients with cystic fibrosis (CF) [3] belong to the most important antibiotic-resistant “priority pathogens” classified by the WHO [4]. This opportunistic pathogen is responsible for severe and often deadly infections in immunocompromised patients. Moreover, with the recent increase in the life span of CF patients, new pathogens have become clinically relevant, especially non-tuberculous mycobacteria such as *Mycobacterium abscessus* (*M.abs*.) [5]. Notably, *M.abs*. is responsible for cutaneous and pulmonary infections, representing up to 95% of non-tuberculous mycobacteria infections in CF patients, and is one of the most drug-resistant mycobacteria for which standardized chemotherapeutic regimens are still lacking [6]. Thus, the search continues for new effective antimicrobial compounds.

First antimicrobial peptides (AMPs) were discovered in the early ’80s and they are usually described as <100 amino acids in length, amphipathic, and cationic molecules with potential to kill microbes [7,8]. The very few AMPs in clinical trials are analogs or derivatives of natural AMP sequences from various animal species (humans, amphibians, insects). The multi-target interaction of AMPs with the bacterial membrane minimizes the induction of multidrug resistance (MDR) [9] and their antibacterial activity against biofilms also adds value to AMPs compared to classic antibiotics [10,11]. However, their low stability in the bloodstream has limited their clinical application until now.

Thirty years after their discovery, AMPs “stage a comeback” due to a better understanding of their mode of action and remarkable progress in their synthesis and modification methods [12]. For example, the halictines (HAL-1 and HAL-2) have been isolated from the venom of honey bees [13]. Recently, a series of Hal-2 analogs were synthesized and characterized to identify peptides retaining high antimicrobial activity while exhibiting lower toxicity to eukaryotic cells than the original HAL-2, revealing the promising GKWMKLLKKILK-NH_2_ (PEP) sequence which shows high antimicrobial activity and low toxicity to eukaryotic cells [14].

Effective antibacterial therapy requires the successful delivery of antimicrobial agents to the infected site in a sufficient amount. Polymer-based drug delivery systems (DDS) can offer prolonged drug circulation, protection against degradation in the circulation, active targeting of specific cells, and thus reduced toxicity against healthy tissues. The use of nano-sized DDS is very promising for enhanced, side-effect-free, and specific treatment of various diseases, including neoplastic, inflammatory, and also infectious diseases [15], for example, biocompatible, non-toxic, and non-immunogenic copolymers based on *N*-(2-hydroxypropyl)methacrylamide (HPMA) were studied thoroughly in this regards. These water-soluble polymer-carriers bear low-molecular-weight drugs or oligopeptides, e.g., cytostatic, anti-inflammatory, or antimicrobial agents, covalently bound to the polymer backbone via biodegradable spacers, designed for controlled release of the active molecules in infected cells, organs and tissues [15]. Compared to free drugs, the polymer-drug conjugates display superior activity with improved pharmacokinetic properties, enhanced stability, targeting ability, and controlled activation at the desired site [16]. The active molecules can be released by reductive degradation, enzymatic hydrolysis, or by pH-dependent hydrolysis triggered by a decrease of pH from 7.4 (blood) to 5–6 (endosomes/lysosomes). If the active substances are biologically active peptides or proteins, their modification with water-soluble HPMA copolymers can also significantly improve their stability in the systemic body circulation. Recently, polymer conjugates of superoxide dismutase (SOD) were demonstrated to be highly stable nanosystems [17]. It is believed that nano-sized delivery systems with antibiotics might improve the treatment of patients with various infectious diseases, thereby overcoming the severe global burden of antibiotic resistance [18].

Herein, we describe the rational design, synthesis, and preliminary antibacterial evaluation of polymer-antibacterial peptide GKWMKLLKKILK-NH_2_ conjugates. These macromolecular constructs were designed as stimuli-sensitive drug delivery systems to protect the AMP during its delivery to the bacterial infection site where the free AMP is released by enzymatic or pH-sensitive hydrolysis. The rate of the AMP release is controlled by the structure of the linker between the AMP and the HPMA-based polymer carrier.

## 2. Materials and Methods

### 2.1. Materials

1-Aminopropan-2-ol, 2,2’-azobis-(isobutyronitrile) (AIBN), dichloromethane (DCM), *N*, *N*′-diisopropylcarbodiimide (DIC), *N, N*’-dimethylacetamide (DMA), *N*, *N*′-dimethylformamide (DMF), ethyldiisopropylamine (DIPEA), hydrazine monohydrate, methacryloyl chloride, triisopropylsilane (TIPS) and all other reagents and solvents were purchased from Sigma-Aldrich (Sigma-Aldrich, Prague, Czech Republic). Chain-transfer agents and initiators were purchased from FUJIFILM Wako Pure Chemical Corporation (FUJIFILM Wako Pure Chemical Corporation, Neuss, Germany). TentaGel Rink amide resin, ethyl cyano(hydroxyimino)acetate (Oxyma), (benzotriazol-1-yloxy)-trispyrrolidinophosphoniumhexafluorophosphate (PyBOP), trifluoroacetic acid (TFA), 9-fluorenylmethyloxycarbonyl (Fmoc)-amino acid derivatives were purchased from Iris Biotech GmbH (Iris Biotech GmbH, Marktredwitz, Germany). 5-Azidopentanoic acid was obtained from Bachem (Bachem, Bubendorf, Switzerland) and amino-1-(11,12-didehydrodibenzo[b,f]azocin-5(6H)-yl)propan-1-one (DBCO-NH_2_) was purchased from Click Chemistry Tools (Click Chemistry Tools, Scottsdale, AZ, USA). ATTO-488-NHS and ATTO-488-NH_2_ were purchased from ATTO-TEC GmbH (ATTO-TEC GmbH, Siegen, Germany).

### 2.2. Methods

Purity of the AMP peptides, conjugation of dyes or antimicrobial peptides to polymer precursors was monitored by HPLC using a Chromolith Performance RP-18e column (100 × 4.6 mm; Merck, Darmstadt, Germany) using a linear gradient of water-acetonitrile, 0–100% acetonitrile in the presence of 0.1% TFA with a UV-vis diode array detector (Shimadzu, Kyoto, Japan). The amino-acid analysis of the hydrolyzed samples (6 M HCl, 115 °C, 18 h in a flame-sealed ampule) was performed on the same Chromolith Performance RP-18e reversed-phase column by pre-column derivatization with *o*-phthalaldehyde and 3-sulphanylpropanoic acid (excitation at 229 nm, emission at 450 nm) and gradient elution with 10–100% of solvent B for 35 min at a flow rate of 1.0 mL‧min^−1^ (solvent A: 0.05 M sodium acetate buffer, pH 6.5; solvent B: 300 mL of 0.17 M sodium acetate and 700 mL of MeOH).

The molecular weights and dispersity (*Ð*) of the polymer precursors were determined by size-exclusion chromatography (SEC) on an HPLC system (Shimadzu) equipped with a refractive index, UV, and multiangle light scattering DAWN 8 EOS (Wyatt Technology Corp., Santa Barbara, CA) detectors using a TSK 3000 SWXL column (Tosoh Bioscience, Tokyo, Japan) and 80% MeOH:20% 0.3 M acetate buffer pH 6.5 at a flow rate of 0.5 mL‧min^−1^. The calculation of molecular weights from the light scattering detector was based on the known injected mass assuming 100% mass recovery and RI detector with the refractive index increment d*n*/d*c* equal to 0.167 mL‧g^−1^. The PEP release was also measured by SEC using Superose 12 10/300 GL column (Cytiva, Marlborough, MA, USA) and 0.2M Phosphate-buffered saline (PBS, pH 7.4) as a mobile phase at a flow rate 0.7 mL‧min^−1^.

The molecular mass of the peptide products was determined by mass spectrometry performed on an LCQ Fleet mass analyzer with electrospray ionization (ESI MS) (Thermo Fisher Scientific, Inc., Waltham, MA, USA) or by MALDI TOF spectroscopy using a Bruker Biflex III mass spectrometer (Bruker Daltonics, Billerica, MA, USA).

The content of thiazolidine-2-thione (TT) groups was determined spectrophotometrically on a Helios Alpha UV-vis spectrophotometer (Thermo Fisher Scientific, Oxford, UK) using the molar absorption coefficient for TT in MeOH, ε_305_ = 10 280 L‧mol^−1^‧cm^−1^ and the content of the hydrazide groups was determined by a TNBSA assay [19].

The hydrodynamic diameter (*D*_H_) was measured by dynamic light scattering (DLS) on a Nano-ZS instrument (ZEN3600, Malvern, UK). The samples were dissolved in PBS (1 mg‧mL^−1^) and filtered through a 0.45 μm PVDF filter. The intensity of the scattered light was detected at angle θ = 173° with a laser wavelength of 632.8 nm. The values were determined as a mean ± SD of at least three independent measurements.

### 2.3. Synthesis of Peptide Derivatives

Peptide sequences derived from the basic sequence GKWMKLLKKILK-NH_2_ were assembled by automatic solid phase peptide synthesis using a Liberty Blue microwave peptide synthesizer (CEM, Matthews, NC, USA) starting from the C-terminus using standard Fmoc procedures with the consecutive addition of the *N*-Fmoc-protected amino acid derivative (2.5 equiv.), DIC (2.5 equiv.) as an activator and Oxyma (2.5 equiv.) as an additive, all dissolved in DMF. After attachment of the last *N*-Fmoc-protected amino acid and removal of the Fmoc group, final attachment of levulinic acid or 5-azidopentanoic acid (2.5 equiv.) to form the keto- or azide-peptide derivatives, respectively, was carried out using PyBOP (2.5 equiv.) as an activator and DIPEA (5 equiv.) as an activator base.

**Figure 1:**
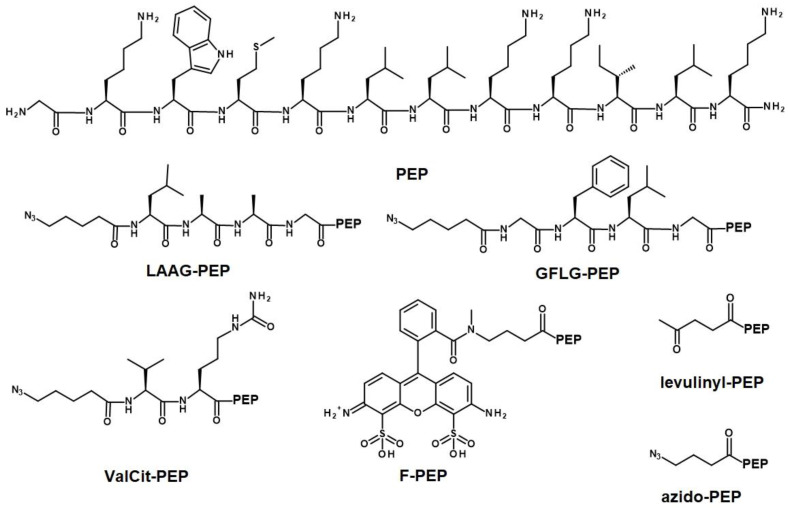
Structures of the antimicrobial peptide **PEP** and prepared peptide derivatives.

The peptide labeled with the dye ATTO-488 (F-PEP), was prepared as follows. After the final Fmoc removal from the peptide-resin, ATTO-488-NHS (5 mg, 5.1 µmol) in DMA (250 µL) was added to the peptide-resin (20 mg). After 2 h, the resin was washed with DMA, DCM, and MeOH and dried. The peptide was cleaved from the resin using a mixture of 95% TFA, 2.5% TIPS, and 2.5% water (200 µL) for 1 h. The solution was evaporated, and the peptide was dissolved in water/dioxane (1/1 *v*/*v*) and lyophilized. The structures of all peptide derivatives are shown in Figure 1 and were characterized using HPLC and ESI MS (levulinyl-PEP: calculated 1582.1 g‧mol^−1^, found 791.0 g‧mol^−1^, M/2; azido-PEP: calculated 1609.0 g‧mol^−1^, found 804.33 g‧mol^−1^, M/2; LAAG-PEP: calculated 1921.2 g‧mol^−1^, found 1922.16 g‧mol^−1^, M + H; GFLG-PEP: calculated 1983.2 g‧mol^−1^, found 1984.16 g‧mol^−1^, M + H; ValCit-PEP: calculated 1864.2 g‧mol^−1^, found 931.75 g‧mol^−1^, M/2; and F-PEP: calculated: 2056.1 g‧mol^−1^, found 2057.32 g‧mol^−1^, M + H). Additional information in Supplementary Materials as Figures S1–S13.

### 2.4. Synthesis of the Monomers

HPMA was prepared by the reaction of methacryloyl chloride with 1-aminopropan-2-ol in dichloromethane (DCM) as described earlier [20]. 3-(3-Methacrylamidopropanoyl)thiazolidine-2-thione (Ma-β-Ala-TT) was prepared by two-step procedure. First, 3-methacrylamidopropanoic acid (Ma-β-Ala-OH) was synthesized by the reaction of methacryloyl chloride with 3-aminopropanoic acid in an aqueous alkaline medium. In the second step, the reaction of Ma-β-Ala-OH with 1,3-thiazolidine-2-thione was carried out according to the literature [21]. 1-(*tert*-butoxycarbonyl)-2-(6-methacryl-amidohexanoyl)hydrazine (Ma-Acap-NHNH-Boc) was prepared as described previously [22].

### 2.5. Synthesis of the Polymer Precursor

Amino-reactive polymer **1**, p(HPMA-*co*-Ma-β-Ala-TT), was prepared by RAFT polymerization of HPMA (90 mol%, 1 g, 6.98 mmol) and Ma-β-Ala-TT (10 mol%, 200 mg, 0.78 mmol) using AIBN (2.3 mg, 14.1 μmol) as an initiator and 2-cyanopropan-2-yl benzenecarbodithioate (6.3 mg, 28.2 μmol) as a chain-transfer agent (CTA) as described previously [23].

Polymer **2**, p(HPMA-*co*-Ma-β-Ala-DBCO), intended for copper-free click chemistry was prepared by the aminolytic modification of polymer **1** as follows: polymer **1** (135 mg, 92.9 μmol of TT groups) and 9.6 mg DBCO-NH_2_ (34.7 μmol, equivalent to 4 mol% TT groups) were dissolved in 1.4 mL DMA and 8 µL of DIPEA (46.7 μmol) was added and the reaction was stirred overnight. The remaining TT groups on the polymer were removed by the addition of 1-aminopropan-2-ol (7.2 μL, 94 μmol) and polymer **2** was precipitated into acetone/diethyl ether (1/1 *v*/*v*), washed with diethyl ether, and dried.

Polymer **3**, p(HPMA-Ma-Acap-NHNH_2_), with hydrazide groups along the chain was prepared by RAFT polymerization of HPMA (88 mol%, 150 mg, 1.05 mmol) and Ma-Acap-NHNH-Boc (12 mol%, 45 mg, 0.14 mmol) using 2,2’-azobis(4-methoxy-2,4-dimethylvaleronitrile) (V-70; 0.64 mg, 2.09 μmol) as an initiator and *S*-2-Cyano-2-propyl-*S*’-ethyl trithiocarbonate (0.84 mg, 4.18 μmol) as a CTA. HPMA and initiator were dissolved in *t*-BuOH (1.53 mL) and mixed with a solution of Ma-Acap-NHNH-Boc and CTA in DMA (170 µL) to polymerize for 16 h at 40 °C. The reaction mixture was precipitated in acetone/diethyl ether (2/1 *v*/*v*) and re-precipitated from MeOH to obtain 150 mg of polymer precursor **3**. The copolymer was reacted with AIBN (10 molar excess) in 3 mL DMA under argon atmosphere for 2 h at 70 °C in a sealed ampule to remove trithiocarbonate ω-end groups [24]. The reaction mixture was isolated by precipitation to diethyl ether, the precipitate was washed with diethyl ether and dried under a vacuum. To remove the Boc-protecting group, the polymer was dissolved in 1.5 mL solution of 95% TFA, 2.5% TIPS, and 2.5% water and after 1 h precipitated in diethyl ether, washed with diethyl ether and dried under vacuum to yield copolymer **3** (140 mg, yield 72%).

Fluorescently labeled polymer **4,** p(HPMA-*co*-Ma-β-Ala-ATTO-488-*co*-Ma-β-Ala-DBCO), was prepared by the reaction of polymer **1** (98 mg, 10.7 mol% of TT groups, 67.4 μmol of TT) and ATTO-488-NH_2_ (2 mg, 2.3 µmol) in 1 mL DMA in presence of 5 eqiv. DIPEA related to the dye. After two hours, DBCO-NH_2_ (7.1 mg, 25.7 µmol, equivalent to 4 mol% TT groups) was added with DIPEA (6 µL) and the reaction was stirred overnight. The remaining TT groups were removed by the addition of 1-aminopropan-2-ol (5.3 µL, 69.4 μmol); the crude product was precipitated into acetone/diethyl ether (1/1 *v*/*v*), washed with diethyl ether, and dried to yield 73 mg of fluorescently labeled polymer **4.**

### 2.6. Synthesis of the Polymer Conjugates with Azido-Peptides

Polymer **2** (85 mg, 4 mol% of DBCO groups) and azido-peptide, i.e., **azide-PEP**, **azide-ValCit-PEP**, **azide-LAAG-PEP** or **azide-GFLG-PEP**, (15 mg, 15 wt%) were dissolved in DMA (1 mL). The course of the click reaction was monitored by HPLC. After 2 h, no free peptide was found within the reaction mixture and the solution was precipitated into acetone/diethyl ether (1/1 *v*/*v*), washed with diethyl ether, and dried. The fluorescently labeled polymer construct **F-P-PEP** was prepared analogously from the polymer precursor **4**. The reactions were monitored by HPLC and there was no free peptide after 20 h. All the polymer-peptide constructs were precipitated into acetone/diethyl ether (1/1 *v*/*v*), washed with diethyl ether, and dried. The polymer constructs were dissolved in water, purified by chromatography on Sephadex G-25 in water (PD10 column, Cytiva), and freeze-dried.

### 2.7. Synthesis of the Polymer **P-Hyd-PEP** with Keto Group Containing **Levulinyl-PEP**

Polymer (85 mg, 5.9 mol% of hydrazide groups) was dissolved in MeOH (740 µL) and mixed with a solution of **levulinyl-PEP** (15 mg, 15 wt%) in MeOH (100 µL) with acetic acid (35 µL). The reaction was stirred for 48 h and then precipitated into acetone/diethyl ether (1/1 *v*/*v*), washed with diethyl ether, and dried to yield 75 % of the polymer.

### 2.8. Stability and Release of the PEP

The enzymatic activity of cathepsin B was probed immediately before the release measurements as described previously [23]. The enzymolysis was monitored by HPLC and the rate of **PEP** release was calculated from the area of the corresponding polymer peaks from the fluorescent detector (excitation 280 nm, emission 350 nm of Trp in PEP). The spontaneous hydrolysis and release of **PEP** were measured by HPLC in two different PBS buffers to mimic the bloodstream, pH 7.4 and pH 5.5, at 37 °C.

### 2.9. Determination of Minimal Inhibitory Concentration (MIC) and Minimum Bactericidal Concentration (MBC)

The antibacterial properties of HPMA-PEP constructs were tested against *Acinetobacter baumanii* (CCM 2355), *Escherichia coli* (CCM 4517), *Staphylococcus epidermidis* (CCM 2124), and *Staphylococcus aureus* (CCM 4516) (Czech Collection of Microorganisms, Brno, Czech Republic) in vitro. A single colony of the test microorganism was transferred into 5 mL of sterile broth (Luria-Bertani medium, Sigma) and cultivated overnight at 37 °C to prepare a bacterial test suspension in a double-concentrated Mueller-Hinton broth at a density equivalent to a 0.5 McFarland standard. Desired weight of lyophilized **PEP** or polymer-PEP was resuspended in sterile PBS and a concentration series was prepared. The bacterial suspension was then mixed with different concentrations of the **PEP** or polymer-PEP construct diluted in 1:1 in PBS and cultured at 35 °C. The bacterial growth was measured spectrophotometrically at 625 nm every 60 min for 20 h using an EON microplate spectrophotometer (Agilent Technologies, Santa Clara, CA, USA).

The MIC was determined as the lowest concentration of **PEP** or polymer-PEP construct without observable bacterial growth. MIC was calculated as the molar concentration of **PEP**. The MBCs were also evaluated by culturing the samples (25 µL in 5 replicates) at MIC on plate count agar at 37 °C. If the bacteria were absent after 24 h of incubation, it was termed MBC.

### 2.10. Transmission Electron Microscopy (TEM)

Bacteria in the exponential growth phase were mixed with the **PEP** or polymer-PEP constructs at a concentration equivalent to 2×MIC. After 2.5 h of incubation, 10 µL of the suspension was placed on a copper carbon-coated electron microscopic copper grid (Sigma Aldrich, Saint Louis, MO, USA) and left to adhere for 10 min. Then, the grid was washed twice with dH_2_O and stained with 0.8% sodium silicotungstate (Agar Scientific, Stansted, UK), pH 7.4, for 40 s. The samples were analyzed using a JEOL JEM-1010 instrument (JEOL Ltd., Tokyo, Japan) with an SIS MegaView III digital camera (Soft Imaging Systems) at acceleration voltage 80 kV and AnalySIS Software 2.0.

### 2.11. Fluorescent Microscopy

The densities of the fresh overnight bacterial cultures were adjusted to the 0.5 McFarland standard, and 100 µL was placed in a sterile 96-well plate. The plates were incubated at room temperature for 2 h before the amount of fluorescently labeled **F-PEP** or **F-P-PEP** construct corresponding to its MIC was added to the appropriate wells. Images were captured immediately using a spinning disc confocal microscope (Andor xD revolution) on the Olympus IX81 platform and analyzed with iQ3 software, the excitation wavelength was 488 nm and the emission wavelength was 525 nm.

## 3. Results and Discussion

**Figure 2:**
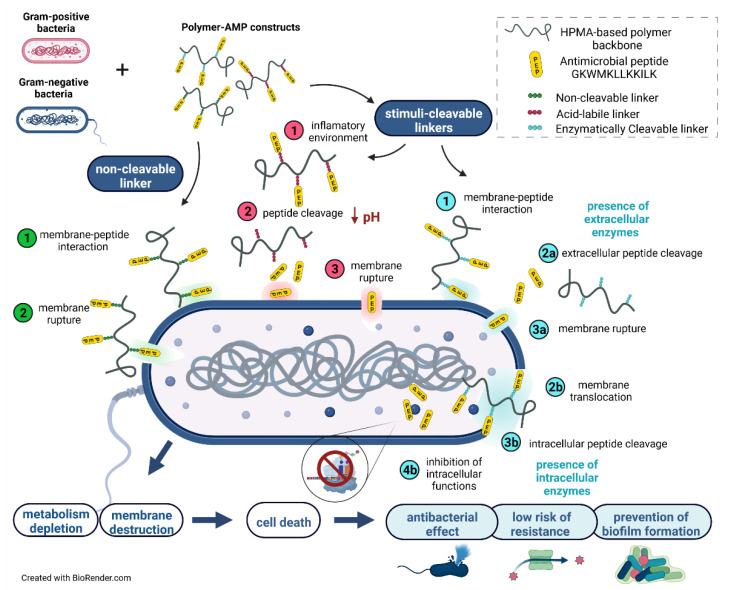
The proposed mechanism of the polymer-antimicrobial peptide construct action. Green—“polymer-bound PEP caused the rupture of bacterial membrane”—the bacterial membrane is disrupted by the polymer-bound AMP. Red—“released PEP caused the rupture of bacterial membrane”—pH-responsive extracellular release of the AMP followed by membrane interaction of low-molecular-weight AMP. Blue—“enzymatically-triggered PEPs’ release and action”—the mechanism is divided into: (i) the enzymatic AMP release caused by extracellular membrane-enzymes, and (ii) polymer-bound AMP penetration of the bacterial membrane, followed by enzymatically triggered cleavage that causes inhibition of various intracellular functions. Figure was created with Biorender.com.

Biocompatible hydrophilic polymers are suitable biomaterials for drug delivery vectors or the hydrophilic coating of nanosized objects, such as nanoparticles, liposomes, or polymerosomes [25]. Due to the excellent biocompatibility of these polymers and their ability to protect the carried molecules from the activity of the reticuloendothelial system, they can be used to protect biologically active peptides and proteins that are usually rapidly degraded in body fluids. Herein, we designed a drug delivery platform for the controlled delivery and activation of AMPs. The platform was based on the covalent attachment of AMP to water-soluble polymers, which are tailored for the specific stimuli-sensitive release of the AMP at the site of bacterial infection, Figure 2.

### 3.1. Synthesis of Polymer Precursors

Controlled RAFT radical polymerization was employed to synthesize well-defined polymer precursors with a very narrow molecular weight distribution (dispersity *Đ* < 1.1) containing appropriate functional groups: polymer **1** with 10.7 mol% of amino-reactive TT groups, polymer **2** with DBCO groups for copper-free click chemistry, and polymer **3** with 6.1 mol% of keto-reactive hydrazides groups. Fluorescently labeled polymer **4** was synthesized by subsequent reaction of the TT groups of polymer **1** with the fluorescent dye ATTO-488-amine and DBCO-amine. RAFT polymerization enabled the synthesis of polymers with the appropriate molecular weight of around 40,000 g‧mol^−1^. All the polymer precursors and their characteristics are shown in Table 1.

The reaction of the TT groups of polymer **1** with amino-containing functional molecules, DBCO-amine or ATTO-488-amine, was monitored by HPLC proving the complete disappearance of the peaks corresponding to the free functional molecules. The modification did not change the molecular weight or the dispersity of the polymers. To avoid any possible cross-reaction of the remaining TT groups, they were removed by the addition of 1-aminopropan-2-ol. Both polymers **2** and **4** containing DBCO groups were used to attach the PEP derivatives via highly selective copper-free Strain-Promoted Alkyne-Azide click chemistry.

Polymer **3** was prepared by copolymerization of *N*-Boc-protected hydrazide comonomer Ma-Acap-NHNH-Boc with HPMA to avoid any undesired interference of the unprotected hydrazide groups with the RAFT polymerization components. It has been observed that RAFT polymerization of comonomers containing unprotected hydrazide or amine groups often resulted in copolymers with higher dispersity and lower end-group functionality. Herein, the use of a Boc-protected comonomer led to the synthesis of polymer **3** with the appropriate molecular weight and dispersity. The hydrazide groups were deprotected before the reaction with the levulinyl-PEP derivative using a TFA solution. Importantly, all the polymer precursors showed hydrodynamic diameters around 7.6–8.5 nm ensuring the prolonged blood circulation and subsequent elimination via renal filtration, thus were suitable precursors for the building of the polymer-AMP constructs. Physicochemical characterization of all prepared polymer-PEP constructs is summarized in Table 2.

### 3.2. Synthesis of Polymer-Peptide Constructs

**Figure 3:**
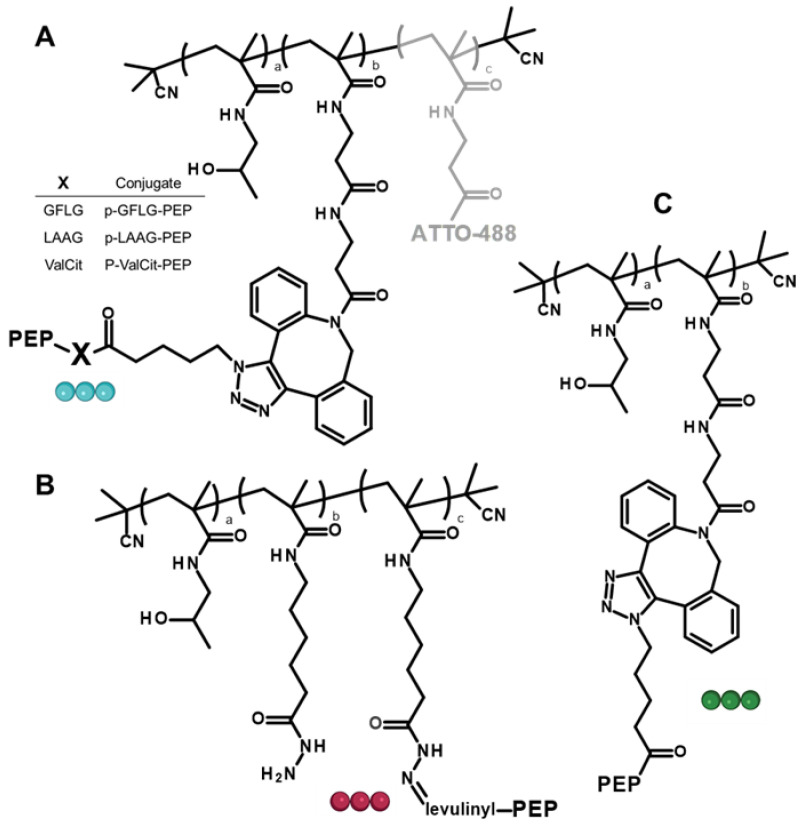
Structures of the polymer-AMP constructs: (**A**) enzymatically degradable, (**B**) hydrolytically cleavable, and (**C**) with a non-cleavable spacer between the polymer backbone and the AMP. Color of the ball linker is related to the mechanism shown in Figure 2.

The prepared polymers were successfully employed for the controlled synthesis of polymer-AMP construct either by the copper-free click reaction affording non-cleavable constructs **P-PEP**, **F-P-PEP**, enzymatically cleavable **P-ValCit-PEP**, **P-LAAG-PEP**, and **P-GFLG-PEP,** or hydrazone bond formation resulting in **P-Hyd-PEP** (hydrolytically cleavable). Structures of prepared constructs are shown in Figure 3. Microwave-equipped solid phase synthesis allowed the controlled and tailored synthesis of the peptides in a one-pot reaction. The peptides consist of the amino acid sequence of the AMP peptide, GKWMKLLKKILK-NH_2_, a suitable spacer, either oligopeptides LAAG, GFLG or ValCit, non-degradable β-Ala spacer or levulinic acid, and the reactive group, azide or keto group, for attachment to the polymers in the range from 10 to 14.8 wt% of the peptide. Moreover, the molecular weight and the dispersity of the polymer-AMP constructs were not significantly affected by the peptide attachment to the polymers. Importantly, the hydrodynamic size of the polymer-AMP constructs was slightly elevated and all conjugates slightly exceeded 10 nm in size. It was hypothesized that such an increase in the size after peptide attachment was due to the repulsive forces of the charged peptides on the hydrophilic uncharged polymer backbone, thereby expanding the polymer random coil and increasing the hydrodynamic size, important behavior from a pharmacokinetic perspective [26]. The increased size of the polymer-AMP construct can prolong the circulation since the renal filtration will be lowered until the peptide is released and the hydrodynamic size of the polymer will decrease enabling glomerular filtration. Importantly, high molecular weight polymer systems are passively accumulated in the inflammation sites [27], thus enhanced polymer-AMP accumulation is expected in the inflamed bacterial infection site.

### 3.3. Design of the Polymer-AMP Constructs, AMP Release, and Stability

**Figure 4:**
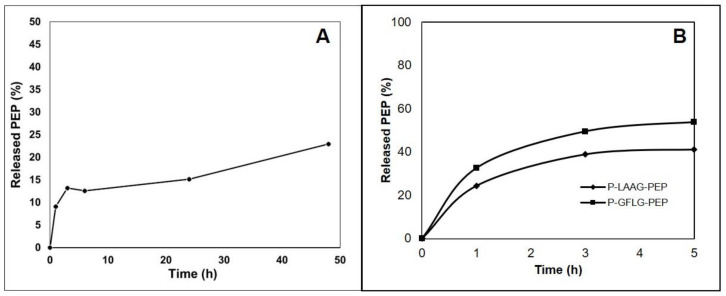
(**A**) Hydrolytic release of **levulinyl-PEP** from the polymer conjugate **P-Hyd-PEP** at pH 7.4. (**B**) Cathepsin B-catalyzed release of PEP from the polymer conjugates.

The polymer-AMP constructs were designed as stimuli-responsive polymer nanotherapeutics for the prolonged blood circulation and consequent site-specific enzymatic and pH-sensitive activation of the AMP. The extracellular protease Staphopain B was thought to activate AMP based on the known composition of the *S. aureus* cell wall [28,29]. The LAAG and GFLG sequences were chosen as suitable biodegradable spacers between the PEP and the polymer carrier [29], intracellular cleavage of which would release the PEP. Moreover, the nondegradable β-Ala spacer was employed in a stable **P-PEP** construct as the control system. Recently, the ValCit spacer was introduced as a suitable spacer for the controlled release of the cytostatics [30], thus it was also applied in this study. Since bacterial infection and biofilm formation may lower the pH in the infected site, a pH-responsive hydrazone bond was introduced as well into the polymer-AMP construct to investigate the benefits of pH-sensitive release and re-activation of the AMP (Figure 4A).

The polymer-AMP constructs were studied in detail for their ability to release the AMP in the presence of proteases and different pH. Unfortunately, Staphopain B production was discontinued, so cathepsin B was used instead. There was approximately 50% PEP release within the first 5 h of the incubation of polymer-AMP constructs with LAAG or GFLG oligopeptide spacer as shown in Figure 4B. After this time, the PEP was subject to degradation so it was not possible to calculate the PEP release rate in the presence of the cathepsin B for longer incubation times. Nevertheless, both tetrapeptide spacers, LAAG and GFLG, are susceptible to enzymatic degradation, thereby we summarize that the PEP bound via the tetrapeptide spacers could be reactivated in contact with the bacterial cell wall. Both polymer conjugates, **P-LAAG-PEP** and **P-GFLG-PEP** were stable in model conditions mimicking the bloodstream, PBS buffer pH 7.4, and no release of the PEP was observed. On the contrary, the ValCit spacer containing conjugate **P-ValCit-PEP** was not releasing the PEP in the contact with cathepsin B, thus this spacer was not recognized as suitable candidate for further investigation. 

Importantly, **P-Hyd-PEP** was relatively stable at pH 7.4, releasing up to 15% of PEP within 24 h of incubation, see Figure 4A. Unfortunately, it was not possible to determine the amount of released PEP at pH 5 due to the HPLC analytical technique and the non-specific interaction of the released PEP. Nevertheless, the pH-sensitive behavior of the hydrazone bond has been proved previously [24,27], thus the PEP release is expected to be pH-dependent. Importantly, the **P-PEP** conjugate with non-degradable β-Ala spacer was stable at both pH 7.4 and pH 5.0 showing no PEP liberation.

### 3.4. Minimum Inhibitory Concentration (MIC) of PEP and PEP-Polymer Constructs

The MICs of PEP and its polymer constructs were determined for four bacterial strains *S. aureus*, *S. epidermidis* (both gram-positive), *E. coli*, and *A. baumanii* (both gram-negative). The MIC of **PEP** was comparable to previously published results [31]. PHPMA alone did not show any growth inhibition (data not shown) in line with its well-known biocompatibility. *S. aureus* was resistant to all polymer-PEP constructs except **P-Hyd-PEP** (see Table 3), possibly due to the extracellular matrix interacting with the polymer constructs thus preventing the antibacterial activity. This was supported by the visualization of bacteria-polymer construct interactions by fluorescent microscopy (see Figure 4). In contrast, the antibacterial properties of **P-Hyd-PEP** were in the same molar range of the MIC as for PEP for all bacterial strains tested, suggesting that the decreased pH during bacterial growth [32] is sufficient to release the PEP attached via a hydrazone bond to the polymer carrier. **P-PEP** also showed antibacterial properties against the remaining three bacterial strains but the MIC was higher than for free PEP. The reduced antibacterial efficacy of **P-PEP** in respect to free PEP is caused by steric hindrance of the PEP in the random coil of the polymer carrier. If the PEP covalently bound to the polymer is not sufficiently exposed before insertion into the bacterial membrane, the efficacy of the membrane disruption is decreased. The same pattern of a higher MIC and lower antibacterial efficacy was also observed for **P-ValCit-PEP**, **P-LAAG-PEP**, and **P-GFLG-PEP**, indicating that the oligopeptide spacers designed as enzymatically degradable sequences did not bring any significant advantage to the efficacy of polymer-PEP constructs. Steric hindrance influences the cleavage of the oligopeptides by the membrane-associated enzyme, thus, the extracellular mechanism of the **P-ValCit-PEP**, **P-LAAG-PEP**, and **P-GFLG-PEP** is the same as for the non-degradable **P-PEP**. Furthermore, the proposed mechanism of intracellular PEP release and action proposed in Figure 2 does not seem to be employed by the PEP.

The gram-positive bacterium *S. epidermidis* showed complete resistance to **P-ValCit-PEP** within the concentration range tested. All tested polymer-PEP constructs were more effective against gram-negative bacteria than gram-positive bacteria probably due to the difference in bacterial surface charge. Gram-negative and Gram-positive bacteria possess a negative charge but gram-negative bacteria are also covered in long polyanionic lipopolysaccharide (LPS) chains that impart a significantly higher surface charge density [33], hence, the cationic polymer-PEP constructs may have a higher affinity for the gram-negative bacterium surface. Similarly, Šálek and co-workers [34] found that cationic poly [2-(dimethylamino)ethyl methacrylate-*co*-ethylene dimethacrylate]nanogels provide better bacteriostatic efficiency against the gram-negative *A. baumannii* compared to gram-positive *S. aureus*.

The higher MIC of **F-PEP** and **F-P-PEP** is most probably caused by the presence of the fluorophore, which strongly affects the physiochemical characteristics of PEP or the polymer-PEP construct and consequently, its interactions with the bacterial cell. The MBCs of PEP and the polymer-based PEP constructs were also determined to be equivalent to the MIC for all the bacterial strains.

Figure 2 shows the proposed hypothetical mechanisms of polymer-bound PEP. As PEP has been described to cause bacterial membrane rupture, we intended to verify whether the polymer-bound PEP retains its membrane-rupture activity, or whether it must be cleaved from the polymer carrier to be re-activated. The data indicated that both the released PEP and polymer-bound PEP caused the rupture of the bacterial membrane. It is evident that the mechanism based on the pH-sensitive PEP release is more effective with a stronger antibacterial effect. By contrast, the intracellular PEP release and action are not effective and present for this antimicrobial peptide PEP.

### 3.5. Visualization of the PEP and PEP Construct’s Effect on Selected Bacterial Strains

**Figure 5:**
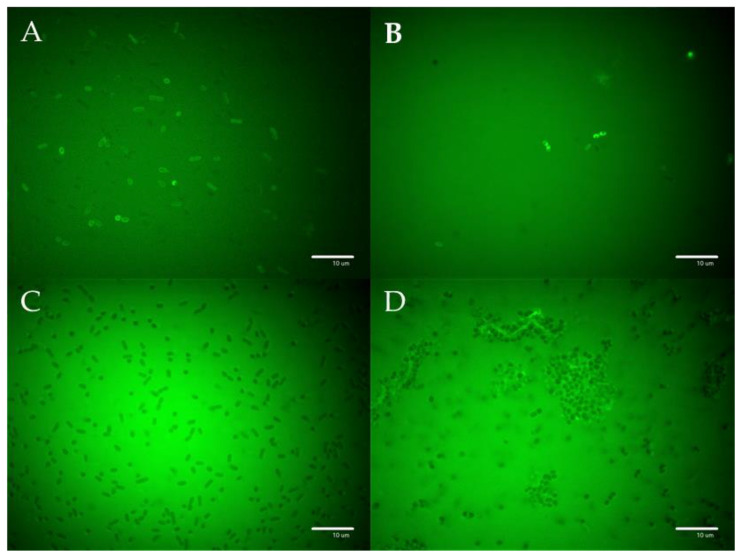
Images of bacterial cells observed using fluorescence microscopy. *E. coli* incubated with (**A**) **F-PEP** (**B**) **F-P-PEP**, and (**C**) **F-P**. Fluorescently-labeled PEP and polymer construct **F-P-PEP** but not the **F-P** polymer showed significant accumulation on the bacterial cell walls. (**D**) **F-P-PEP** interacted with *S. aureus* extracellular structures.

The images acquired after incubation of the fluorescently labeled **F-PEP** and the polymer constructs **F-P** and **F-P-PEP** with either *E. coli* (Figure 5A–C) or *S. aureus* (Figure 5D) are shown in Figure 5. The labeled polymer precursor **F-P** did not interact with the bacterial cells (Figure 5C), whereas there was an accumulation of **F-PEP** and **F-P-PEP** on the bacterial cells, most probably either attached to or immersed in the bacterial cell wall. However, there was considerably less **F-P-PEP** accumulated on *S. aureus* (Figure 5D), supporting the hypothesis that the PEP in **P-PEP** has lower ability to interact with the bacteria wall due to steric hindrance.

**Figure 6:**
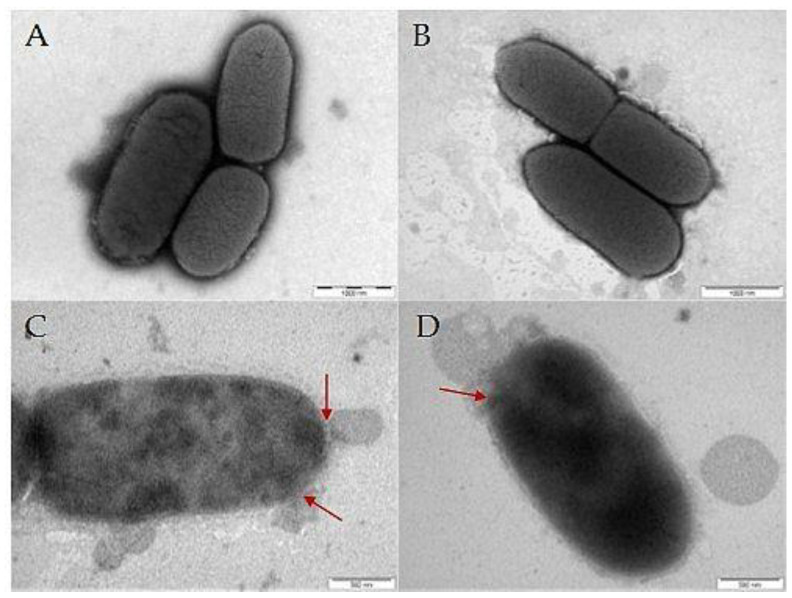
Images of *E. coli* cells obtained by TEM. Disruptions in the bacterial cell wall are highlighted with a red arrow: (**A**) control untreated *E. coli*, (**B**) *E. coli* incubated with control polymer **P,** (**C**) *E. coli* incubated with **PEP**, and (**D**) *E. coli* incubated with **P-PEP**. PEP induced the rupture of the cellular membrane.

Interactions of PEP and polymer-PEP constructs with bacterial cells were also analyzed by TEM. Figure 6A shows the untreated control *E. coli* with a homogenous cytoplasm, and smooth and complete cell wall, and Figure 6B shows that control HPMA-based homopolymer **P** affected neither the bacterial surface nor the cytoplasm. By contrast, *E. coli* treated with 2 × MIC of **PEP** (Figure 6C) or **P-PEP** (Figure 6D) induced cell morphological changes, such as corrugating and roughening of the membrane, disruption of the bacterial cell walls and leakage of the cytoplasm (highlighted with red arrows). These observations further prove the efficacy of the PEP attached to the polymer construct.

**Figure 7:**
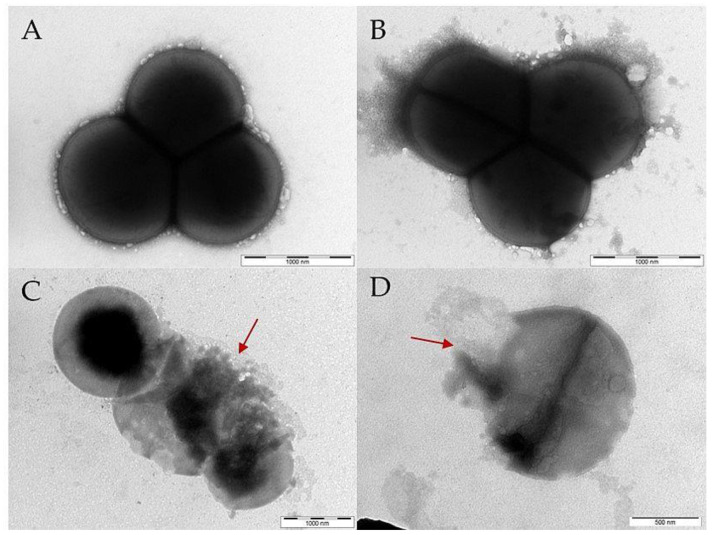
*S. aureus* cells were imaged using TEM. Disruptions in the bacterial cell wall are highlighted with a red arrow: (**A**) control untreated *S. aureus*, (**B**) *S. aureus* treated with the control polymer **P**, (**C**) *S. aureus* incubated with **PEP**, and (**D**) *S. aureus* incubated with **P-PEP**. **PEP** and **P-PEP** caused the rupture of the cell wall.

Similarly, the interaction of PEP and polymer-PEP construct with *S. aureus* was also analyzed by TEM as shown in Figure 7. Likewise, **PEP** (Figure 7C) and **P-PEP** (Figure 7D) treatment of *S. aureus* induced cell morphological changes, cell wall disruption and leakage of the cytoplasm (highlighted by red arrows). In summary, TEM revealed that the PEP attached to the polymer construct destroyed the cell wall of both *E. coli* and *S. aureus*.

## 4. Conclusions

The antimicrobial GKWMKLLKKILK-NH_2_ oligopeptide derived from halictine, honey bee venom, was bound to a hydrophilic biocompatible polymer carrier via various biodegradable spacers sensitive to a change in pH or enzymatically degradable for the release and reactivation of PEP antimicrobial activity. Importantly, PEP retains its antibacterial efficacy against *S. epidermidis*, *E. coli*, and *A. baumanii* even when bound to the polymer carrier. Nevertheless, to obtain remarkable antibacterial activity adequate to the free PEP it is necessary for the peptide PEP to be released from the polymer carrier in response to a pH decrease. The PEP exerts antibacterial activity against gram-positive and negative bacteria via a mechanism that involves disruption of the bacterial cell wall. The presented strategy seems to be highly promising for the delivery of the highly active AMP into the infected parts of the body.

## Figures and Tables

**Table 1:** Characterization of polymer precursors.

Polymer	Structure	Content of Reactive Group mol%	*M*_w_ g‧mol^−1^	*Ð*
**1**	p(HPMA-*co*-Ma-β-Ala-TT)	10.7	40,000	1.06
**2**	p(HPMA-*co*-Ma-β-Ala-DBCO)	4.0	40,800	1.10
**3**	p(HPMA-*co*-Ma-Acap-NHNH_2_)	6.1	39,200	1.05
**4**	p(HPMA-*co*-Ma-β-Ala-ATTO-488-*co*-Ma-β-Ala-DBCO)	4.0	41,200	1.13

**Table 2:** Characterization of the peptide derivatives and polymer-peptide constructs.

Sample	Structure	Precursor	Peptide * wt%	Dye ^#^ wt%	*M*_w_ ^X^ g‧mol^−1^	*Đ* ^X^	*D*_H_ ^XX^ nm
PEP	GKWMKLLKKILK-NH_2_	N/A	100	0	N/A	N/A	N/A
F-PEP	ATTO-488-GKWMKLLKKILK-NH_2_	N/A	72.2	27.8	N/A	N/A	N/A
P	p(HPMA)	N/A	0	0	40,000	1.06	5.9
P-PEP	p(HPMA-*co*-Ma-β-Ala-DBCO-azide-PEP)	2	11.0	0	44,800	1.08	12.6
F-P	p(HPMA-*co*-Ma-β-Ala-ATTO-488)	2	0	2.0	40,000	1.06	8.2
F-P-PEP	p(HPMA-*co*-Ma-β-Ala-ATTO-488-*co*-Ma-β-Ala-DBCO-azide-PEP)	4	11.5	1.6	46,300	1.12	n.d.
P-ValCit-PEP	p(HPMA-*co*-Ma-β-Ala-DBCO-ValCit-PEP)	2	10.0	0	43,300	1.12	12.2
P-LAAG-PEP	p(HPMA-*co*-Ma-β-Ala-DBCO-LAAG-PEP)	2	12.8	0	42,000	1.08	11.2
P-GFLG-PEP	p(HPMA-*co*-Ma-β-Ala-DBCO-GFLG-PEP)	2	11.0	0	41,200	1.09	11.0
P-Hyd-PEP	p(HPMA-*co*-Ma-Acap-NHN=levulinyl-PEP)	3	14.8	0	45,500	1.18	12.6

* Calculated using amino-acid analysis. ^#^ Determined by UV-vis in methanol (ε_505_ = 90,000 L·mol^−1^·cm^−1^). ^X^ Molecular weights were determined by SEC using RI and LS detection. ^XX^ *D*_H_ was measured using DLS (in PBS buffer at 25 °C). N/A non-applicable. n.d. non-determined.

**Table 3:** The MIC (μM) of **PEP** and its polymer constructs. The MIC was determined as the lowest concentration of the construct that inhibited bacterial growth and the data represent the mean of at least three independent experiments.

Bacterial Strain	PEP	P-PEP	P-ValCit-PEP	P-LAAG-PEP	P-GFLG-PEP	P-Hyd-PEP	F-PEP	F-P-PEP
*S.aureus* *	10	-	-	-	-	12.5	72.9	-
*S. epidermidis* *	3.4	14.8	-	172.5	37–74.1	6.3	29.2	77.5
*A. baumanii* †	5	9.3	16.8	10.8–21.6	9.2	6.3	36.5	38.7
*E. coli* †	5	74.1	134	43.1	18.5	6.3	18.2	77.5

* Gram-positive strain. † Gram-negative strain. Dashes indicate that growth inhibition has not been observed within the concentration range tested.

## Data Availability

Not applicable.

## Supplementary Materials

The following supporting information can be downloaded at: https://www.mdpi.com/article/10.3390/pharmaceutics15020406/s1. The Supporting information content: Figure S1: HPLC chromatogram of **levulinyl-PEP**; Figure S2: HPLC chromatogram of **azido-PEP**; Figure S3: HPLC chromatogram of **F-PEP**; Figure S4: HPLC chromatogram of **ValCit-PEP**; Figure S5: HPLC chromatogram of **LAAG-PEP**; Figure S6: HPLC chromatogram of **GFLG-PEP**; Figure S7: The course of the copper-free Strain-Promoted Alkyne-Azide click reaction of **azido-PEP** to the polymer precursor **2**; Figure S8: MS-ESI spectra of **levulinyl-PEP**; Figure S9: MS-ESI spectra of **azido-PEP**; Figure S10: MALDI-TOF spectra of **F-PEP**; Figure S11: MS-ESI spectra of **ValCit-PEP**; Figure S12: MALDI-TOF spectra of **LAAG-PEP**; Figure S13: MALDI-TOF spectra of **GFLG-PEP**.
