# Supplementary material for: Polymer-Antimicrobial Peptide Constructs with Tailored Drug-Release Behavior
Source: Pharmaceutics. 2023 Jan 25;15(2):406. doi: 10.3390/pharmaceutics15020406 (PMC9960778; doi:10.3390/pharmaceutics15020406)
Supplement: Supplementary file 1 [file pharmaceutics-15-00406-s001.zip › pharmaceutics-2132388-supplementary.pdf]

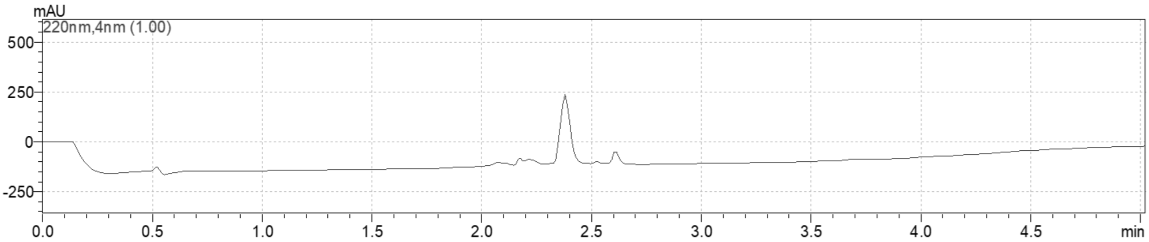

Figure S1: High-performance liquid chromatography (HPLC) chromatogram of **levulinyl-PEP** from PDA detector at 220 nm.

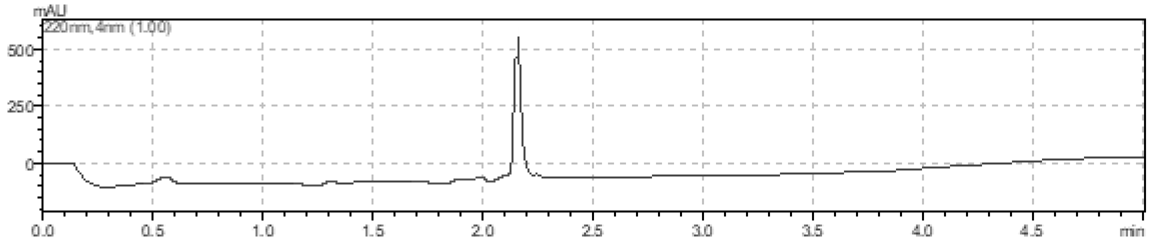

Figure S2: High-performance liquid chromatography (HPLC) chromatogram of **azido-PEP** from PDA detector at 220 nm.

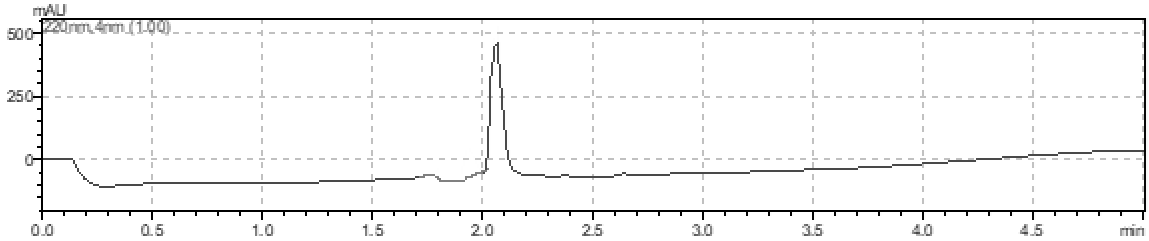

Figure S3: High-performance liquid chromatography (HPLC) chromatogram of **F-PEP** from PDA detector at 220 nm.

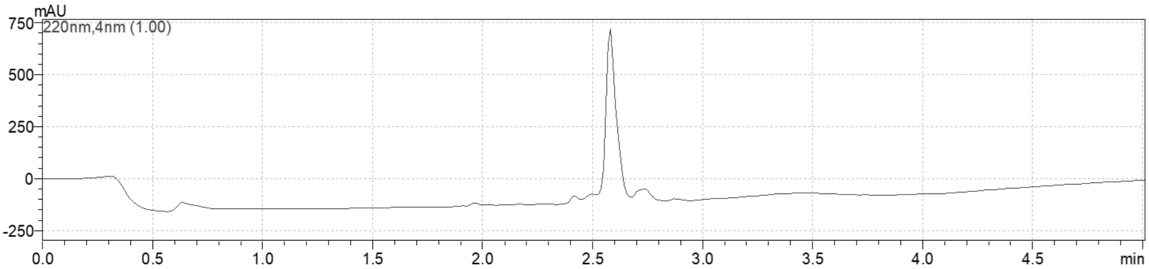

Figure S4: High-performance liquid chromatography (HPLC) chromatogram of **ValCit-PEP** from PDA detector at 220 nm.

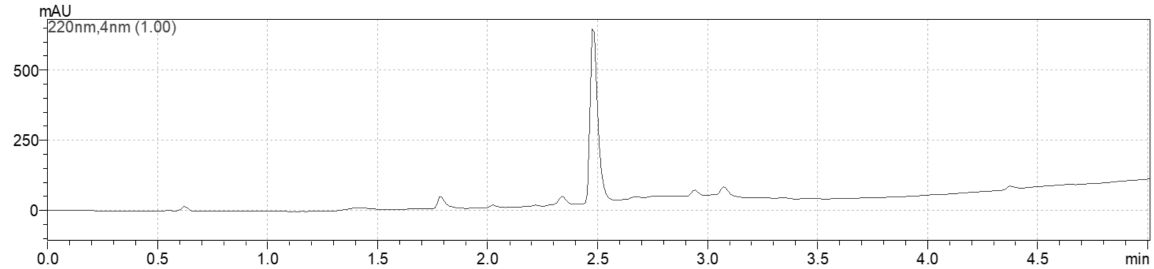

Figure S5: High-performance liquid chromatography (HPLC) chromatogram of **LAAG-PEP** from PDA detector at 220 nm.

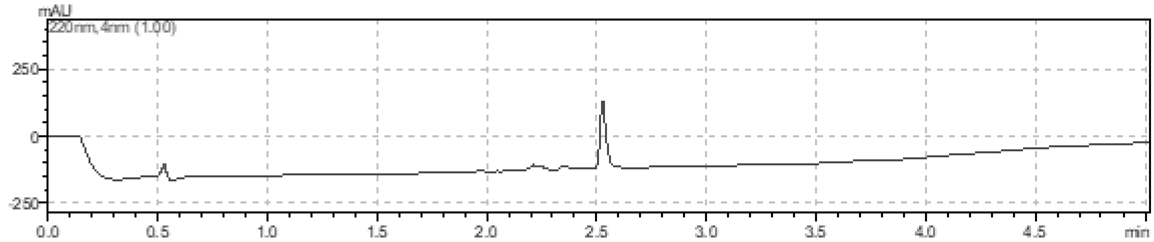

Figure S6: High-performance liquid chromatography (HPLC) chromatogram of **GFLG-PEP** from PDA detector at 220 nm.

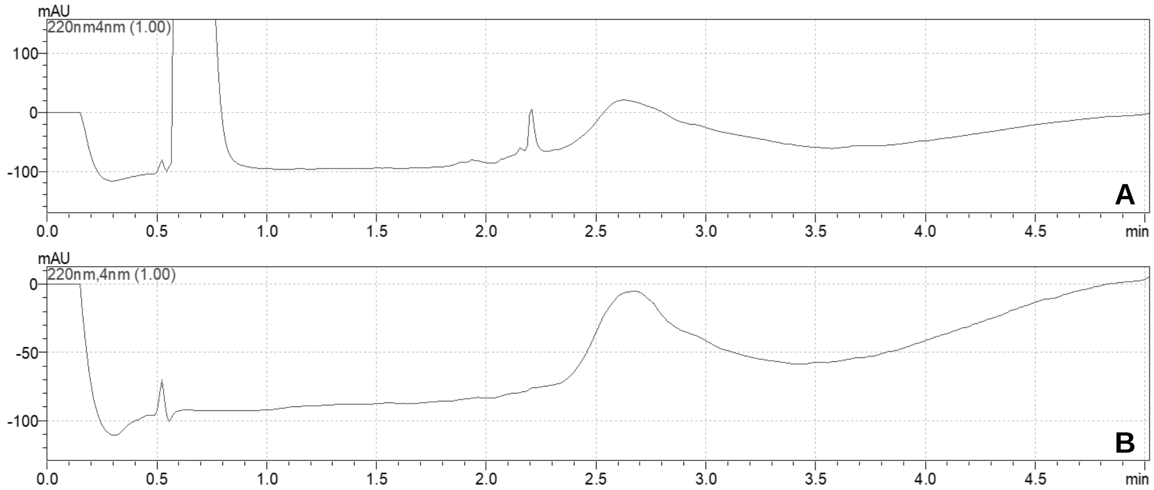

Figure S7: The course of the copper-free Strain-Promoted Alkyne-Azide click reaction of **azido-PEP** to the polymer precursor **2**. (**A**) HPLC chromatogram immediately after mixing in DMA showing the unbound **azido-PEP**, r.t. 2.20 min, and polymer **2**, r.t. 2.65 min. (**B**) HPLC chromatogram of the final polymer conjugate **P-PEP** after purification, no free azide-PEP is present.

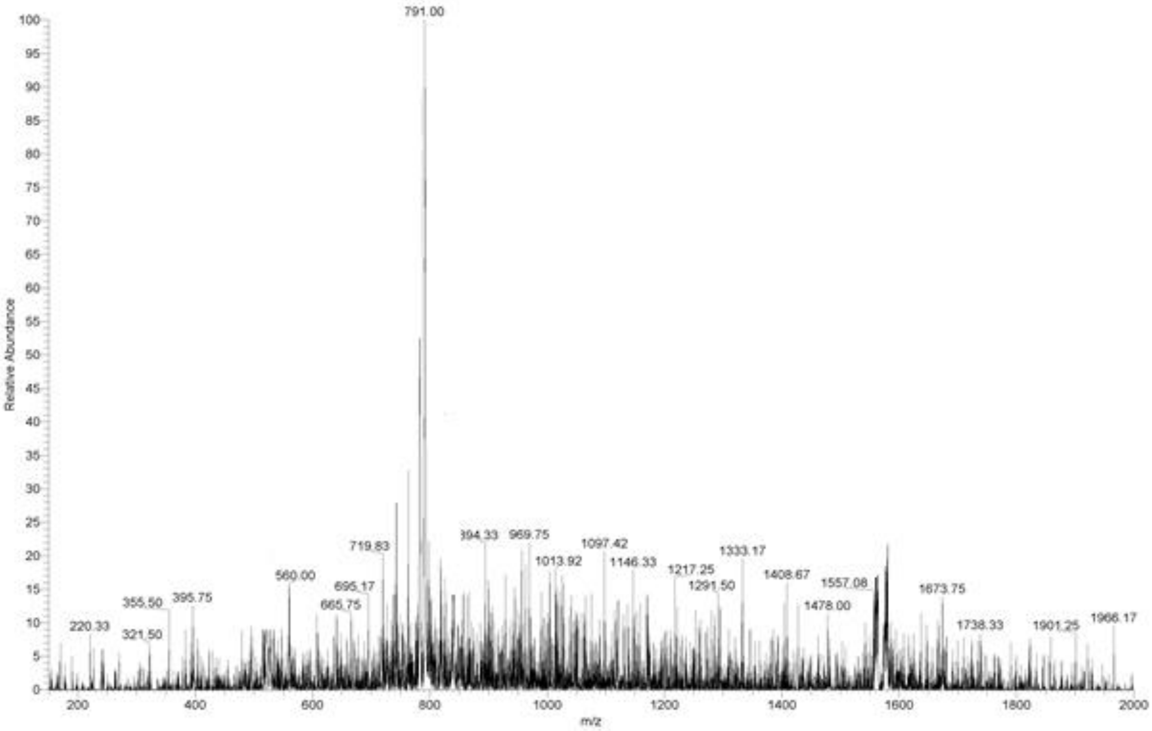

Figure S8: MS-ESI spectra of **levulinyl-PEP** (calculated 1582.1 g·mol^{-1}). Found peak at *m/z 79*1.0 corresponds to M/2 fragment.

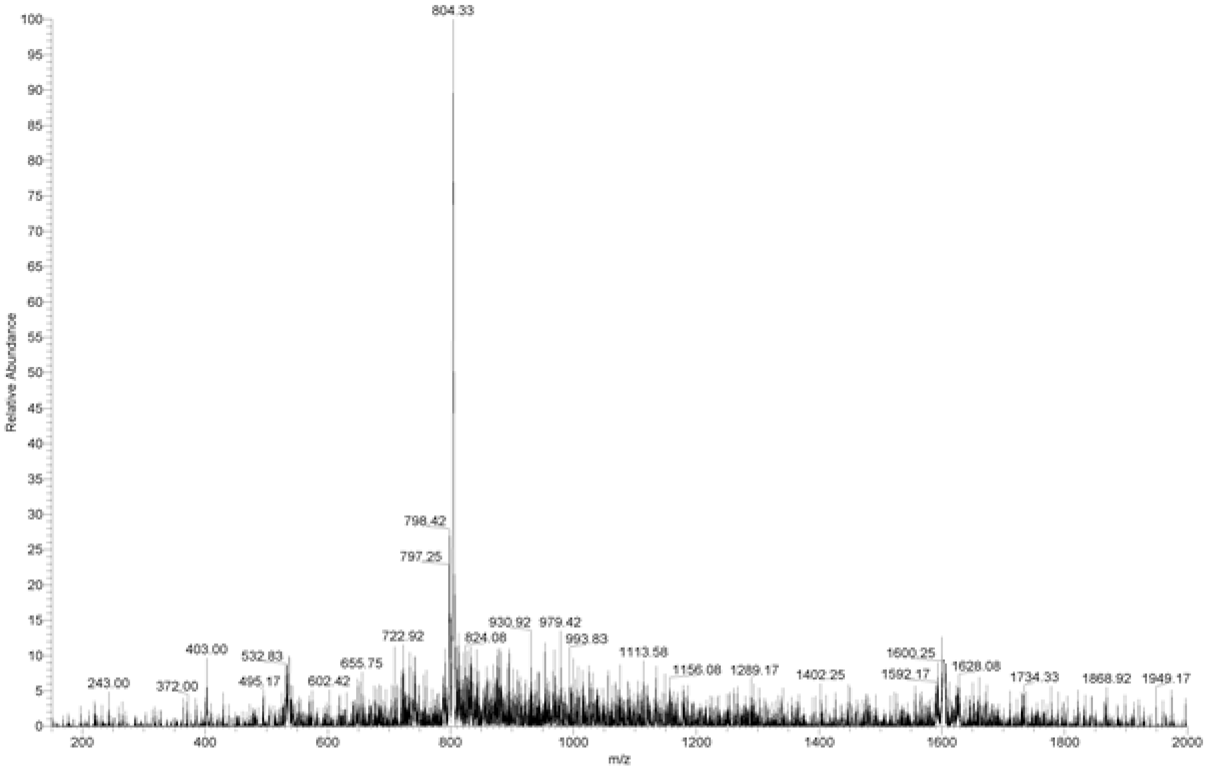

Figure S9: MS-ESI spectra of **azido-PEP** (calculated 1609.1 g·mol^{-1}). Found peak at *m/z 804.*33 corresponds to M/2 fragment.

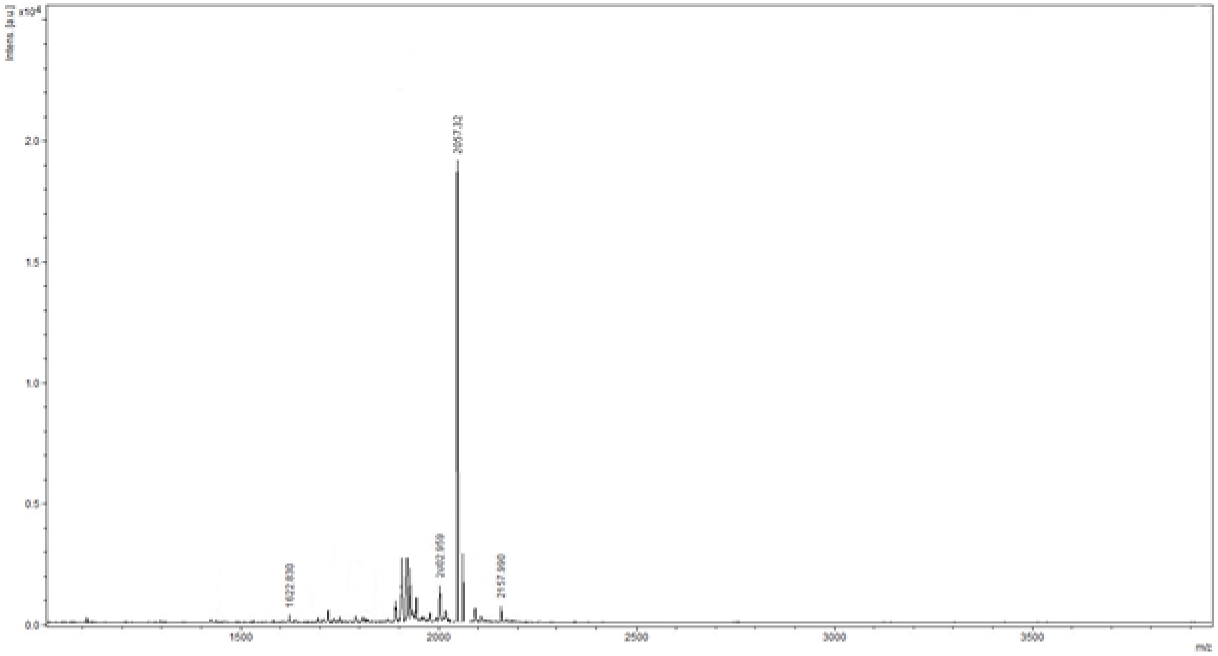

Figure S10: MALDI-TOF spectra of **F-PEP** (calculated 2056.1 g·mol^{-1}, found 2057.32 g·mol^{-1}, M+H).

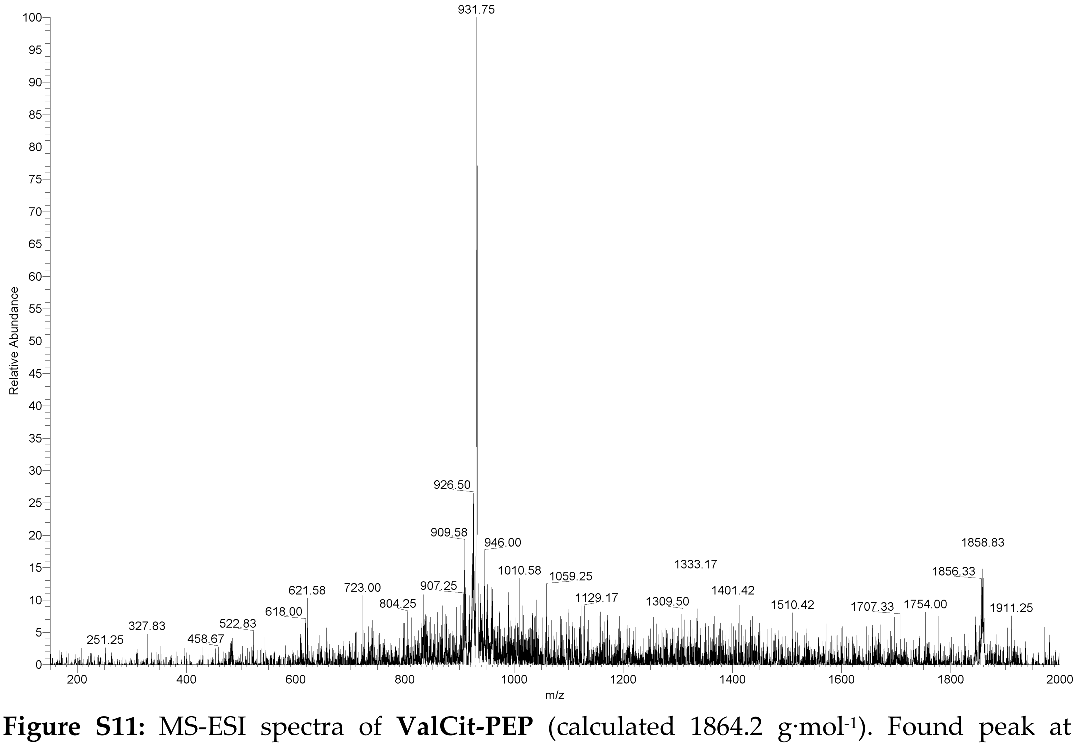

Figure S11: MS-ESI spectra of **ValCit-PEP** (calculated 1864.2 g·mol^{-1}). Found peak at *m/z 931.7*5 corresponds to M/2 fragment

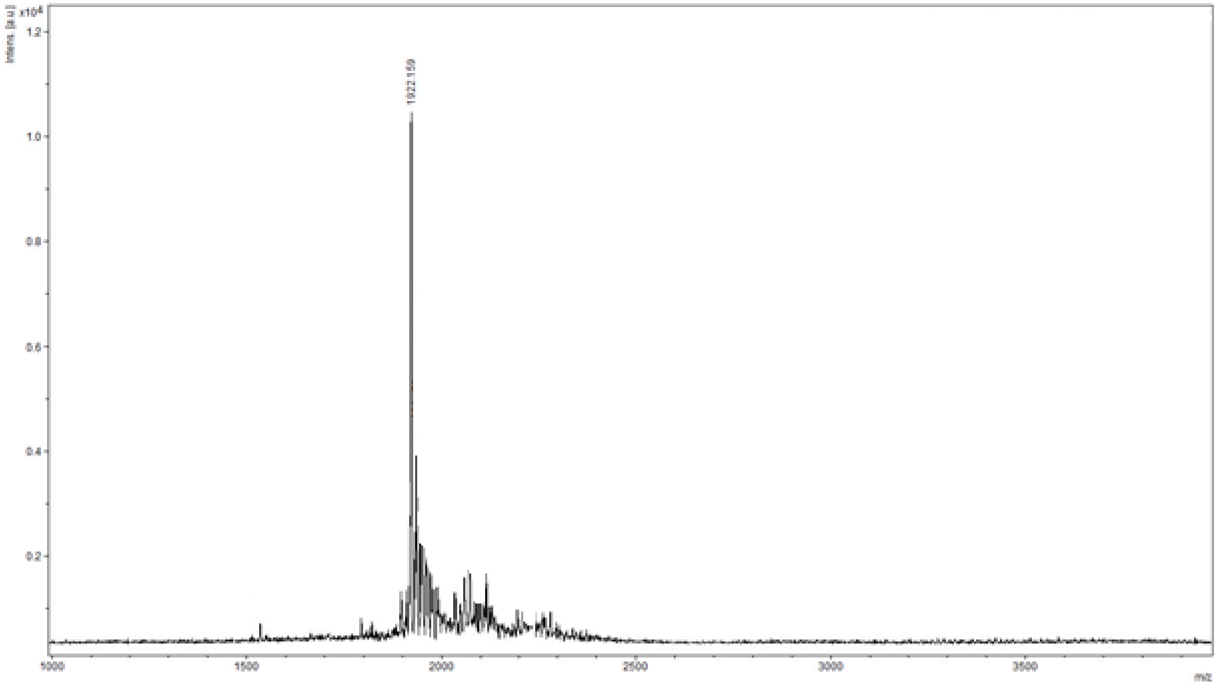

Figure S12: MALDI-TOF spectra of **LAAG-PEP** (calculated 1921.2 g·mol^{-1}, found 1922.16 g·mol^{-1}, M+H).

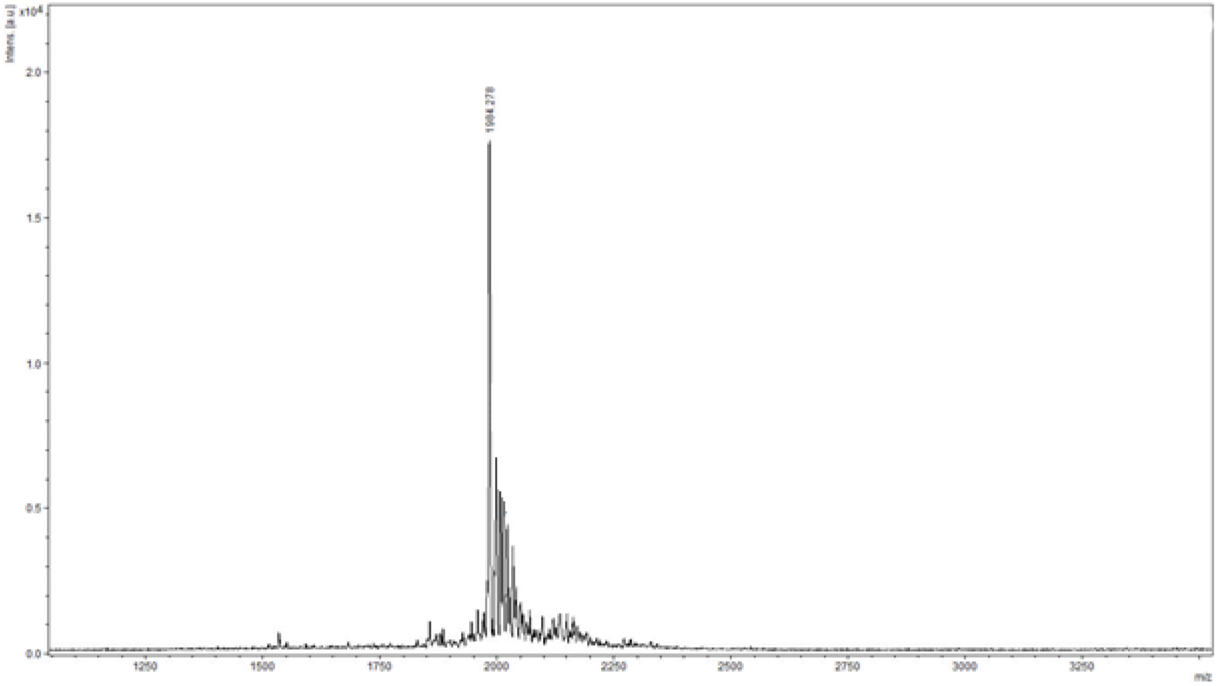

Figure S13: MALDI-TOF spectra of **GFLG-PEP** (calculated 1983.2 g·mol^{-1}, found 1984.16 g·mol^{-1}, M+H).
